# Bond Strengths of Universal Adhesives to Dentin Contaminated with a Hemostatic Agent

**DOI:** 10.3290/j.jad.b3601769

**Published:** 2022-11-23

**Authors:** Sawaphon Noppawong, Jaikaew Pratabsingha, Chanida Thamsoonthorn, Weeranun Vichathai, Pipop Saikaew

**Affiliations:** a Student, Institute of Dentistry, Department of Medical Services, Nonthaburi Thailand. Performed the experiments, wrote the manuscript.; b Student, Institute of Dentistry, Department of Medical Services, Nonthaburi, Thailand. Performed the experiments.; c Lecturer, Institute of Dentistry, Department of Medical Services, Nonthaburi, Thailand. Hypothesis, experimental design, proofread the manuscript.; d Lecturer, Thai Board of General Dentistry, Institute of Dentistry, Department of Medical Services, Nonthaburi, Thailand. Proofread the manuscript.; e Assistant Professor, Department of Operative Dentistry and Endodontics, Faculty of Dentistry, Mahidol University, Bangkok, Thailand. Hypothesis, experimental design, proofread the manuscript, contributed substantially to discussion.

**Keywords:** adhesion to dentin, hemostatic agent, microtensile bond strength, scanning electron microscopy, universal adhesive.

## Abstract

**Purpose:**

To evaluate the microtensile bond strength (µTBS) of three universal adhesives to dentin contaminated with a hemostatic agent.

**Materials and Methods:**

Ninety-six human premolars were cut to expose mid-coronal dentin. The teeth were randomly divided into a control group (uncontaminated dentin) and an experimental group in which a hemostatic agent was applied (contaminated dentin). Each group was further divided into 6 subgroups according to universal adhesives – SBU (Single Bond Universal), OPU (OptiBond Universal), CBQ (Clearfil Universal Bond Quick) – and etching mode, either etch-and-rinse (ER) or self-etch (SE). Following adhesive application, composite was placed in two increments and light cured. The specimens were stored in distilled water at 37°C for 24 h. The µTBS test was performed using a universal testing machine. Failure mode was assessed using a light microscope. The data were statistically analyzed using three-way ANOVA followed by Scheffe’s test (p < 0.05). The resin-dentin interface was observed using scanning electron microscopy.

**Results:**

Significantly lower bond strength was observed when the universal adhesives were bonded to contaminated dentin in SE mode (p < 0.05). In contrast, the µTBS of the universal adhesives in ER mode was not affected by contamination (p > 0.05). The µTBS of CBQ to contaminated dentin was significantly lower than that of the other adhesives. Observation of the resin-dentin interface revealed limited resin penetration when the universal adhesive was applied in SE mode on contaminated dentin.

**Conclusion:**

Contaminating the dentin with a hemostatic agent significantly reduced the µTBS of the universal adhesives in SE mode. However, this adverse effect was not found when the universal adhesives were used in ER mode.

When treating teeth using the adhesive technique in the cervical region, blood and sulcular fluid from the gingiva are an obstacle.^10,26,29^ In addition to applying rubber-dam to isolate the operation field, placing a retraction cord with a hemostatic agent is another preferred method to control moisture and blood contamination. The most commonly used hemostatic agent is aluminum chloride (AlCl_3_), at concentrations between 20%–25%.^5,16^ Ferric sulfate (Fe_2_(SO_4_)_3_) is also used to achieve hemostasis. However, the disadvantage of ferric sulfate is that it can cause gingival tissue staining for several days.^28^

Previous studies reported that the bond strength of dentin contaminated with a hemostatic agent was reduced in self-etch (SE) adhesives.^4,5,9,10,16,18,19,22,26,29^ In contrast, the hemostatic agent did not affect the dentin bond strength when bonded with etch-and-rinse (ER) adhesives.^14,16^ However, most studies compared the bond strength between different adhesives. The different compositions between adhesives could be a confounding factor for the significant differences in bond strength found in these studies.

A universal adhesive has been developed that is less technique sensitive, more user friendly, and more versatile.^21,27^ This adhesive can be applied using either ER or SE mode.^17,20^ The composition of the universal adhesive is similar to contemporary one-step self-etch adhesives. Following the expiration of the 10-methacryloyloxydecyl dihydrogen phosphate (10-MDP) patent, most companies have incorporated 10-MDP into their adhesives, except for OptiBond Universal (Kerr; Orange, CA, USA), which contains glycerol phosphate dimethacrylate (GPDM) as the functional monomer.

Although the effects of hemostatic agents on the bond strength of dental adhesives were evaluated in previous studies, there are few reports on the bond strengths of different universal adhesives containing 10-MDP or GPDM. Information on the newly developed universal adhesive (Clearfil Universal Bond Quick, Kuraray Noritake; Tokyo, Japan) with a reduced application time is particularly limited. Therefore, the aim of this study was to evaluate the µTBS of universal adhesives with different etching modes to human dentin contaminated with an aluminum chloride-containing hemostatic agent. The null hypotheses were that 1) the µTBS of the universal adhesives was not affected by contamination with a hemostatic agent: 2) the µTBS of the universal adhesives was not affected by the etching mode; and 3) there was no difference in µTBS between the 3 universal adhesives.

## MATERIALS AND METHODS

### Tooth Selection and Preparation

The teeth were collected following the approval of the Ethics Review Committee for Research in Human Subjects at the Institute of Dentistry, Department of Medical Services, Thailand (document No. 2/2021). One hundred eight (108) extracted caries-free, fully erupted human premolars were stored in 0.1% thymol at 4°C and used within 6 months after extraction. The teeth were transferred and immersed in 0.9% sodium chloride solution (Otsuka; Samut Sakhon, Thailand) at 4°C and used within 7 days. Flat dentin surfaces were created on the mid-coronal dentin using a low-speed saw (Isomet, Buehler; Lake Bluff, IL, USA) under water cooling, then cleaned in an ultrasonic bath for 10 min. The exposed dentin was inspected with a light microscope (10X) to confirm that all the enamel had been removed and there was no pulpal exposure. The occlusal surface was polished with #600-grit silicon carbide paper (TOA DCC, TOA paint; Samut Prakan, Thailand) under running water for 60 s.

### Adhesives and Bonding Procedures

Ninety-six specimens were used for the bond strength test. Half of the teeth were assigned to the dentin surfaces without contamination (control) group. The remaining teeth were assigned to the contaminated group, in which a hemostatic agent (Racestryptine, Septodont-58; Cedex, France) was applied with a microbrush for 2 min, rinsed with water for 30 s, and air dried for 5 s. The specimens were further divided according to the 3 universal adhesives – Single Bond Universal (SBU, 3M Oral Care; St Paul, MN USA), OptiBond Universal (OPU, Kerr), Clearfil Universal Bond Quick (CBQ, Kuraray Noritake) – and 2 etching modes, self-etch (SE) or etch-and-rinse (ER). The ER groups were etched with Scotchbond Universal Etchant (3M Oral Care) for 15 s, rinsed with water for 15 s, and air dried for 5 s. Next, the adhesive application in all groups was performed according to the manufacturers’ instructions (Table 1). Eight teeth per experimental group were tested as suggested by Armstrong et al.^3^ Following the adhesive application, resin composite (Filtek Z350XT shade A1B, 3M Oral Care) was placed in two increments. Each 2-mm layer was light cured for 20 s using an LED light-curing unit (Elipar DeepCure-L, 3M Oral Care; 1400 mw/cm^2^). The final layer was covered with a glass plate and light finger pressure was applied before a final curing for 40 s. The specimens were stored in distilled water at 37°C for 24 h.

**Table 1:** Chemical compositions of the materials used

Material	pH	Code	Composition	Lot No.	Adhesive application
Single Bond Universal (3M Oral Care; St Paul, MN, USA)	2.7	SBU	10-MDP, HEMA, bis-GMA, dimethacrylate resins, silane, Vitrebond copolymer, fumed silica, ethanol, water, photoinitiators	90913B	Apply adhesive and rub it in for 20 s. Gently air dry for 5 s to evaporate the solvent. Light cure for 10 s.
OptiBond Universal (Kerr; Orange, CA, USA)	2.3	OPU	GPDM, glycerol dimethacrylate, HEMA, acetone, ethanol	7399659	Apply adhesive and rub it in for 20 s. Gently air dry the adhesive for 5 s to evaporate the solvent. Light cure for 10 s.
Clearfil Universal Bond Quick (Kuraray Noritake; Tokyo, Japan)	2.3	CBQ	10-MDP, bis-GMA, ethanol, HEMA, hydrophilic amide monomer, colloidal silica, silane coupling agent, sodium fluoride, camphorquinone, water	7H0266	Apply adhesive and rub it in for 5 s. Gently air dry for >5 s until the adhesive shows no movement. Light cure for 10 s.

10-MDP:10-methacryloyloxydecyl dihydrogenphosphate; HEMA: 2-hydroxyethyl methacrylate; bis-GMA: bisphenol-A glycidyl dimethacrylate; GPDM: glycerol phosphate dimethacrylate.

### Microtensile Bond Strength (µTBS) Test

Each specimen was sectioned into rectangular sticks (cross-sectional area 1 mm × 1 mm) using a low-speed saw. Four resin-dentin sticks per tooth were collected from the central portion of the crown and tested immediately after sectioning. Each resin-dentin stick was fixed to the Ciucchi’s jig with cyanoacrylate glue (Model repair 2 Blue, Dentsply Sirona; Konstanz, Germany) and subjected to a tensile force at a crosshead speed of 1 mm/min using a Lloyd testing machine (Model LR10K, Lloyd Instruments; Fareham, UK). The pre-test failures were recorded as 0 MPa and included in the calculation. The maximum load at failure in N was divided by the bonded area into MPa for the µTBS. The tooth was used as a statistical unit. The data were analyzed for normal distribution using the Kolmogorov-Smirnov test. Three-way ANOVA and Scheffe’s test were calculated using SPSS V26.0 (SPSS; Armonk, NY, USA). The significance level was set at α = 0.05

### Failure Mode Analysis

Following the debonding procedure, the fractured specimens of each subgroup were observed using a light microscope (Nikon Eclipse E-400 Pol with CoolPIX990 screen, Nikon; Tokyo, Japan) at 40X magnification to determine the failure type. The failure was classified as: 1) adhesive; 2) cohesive; or 3) mixed.^11,13^

### Resin-Dentin Interface Observation

Twelve resin-dentin bonded teeth were prepared as described above and randomly assigned to 12 experimental groups. After storing in water at 37°C for 24 h, the resin-dentin slabs of each group were prepared and fixed in epoxy resin. The samples were serially polished with a series of silicon carbide papers (#600–5000 grit) in running water. Next, they were treated with 10% phosphoric acid for 5 s followed by 5.25% NaOCl for 10 min. The resin-dentin slabs were stored in an incubator for 24 h, then coated with palladium (K500X Sputter Coater, SPI Supplies; West Chester, PA, USA). Finally, the resin-dentin interface was observed using a scanning electron microscope (SEM, JSM-6610LV, JEOL; Tokyo, Japan) at 3000X magnification.

## RESULTS

### µTBS

**Fig 1:**
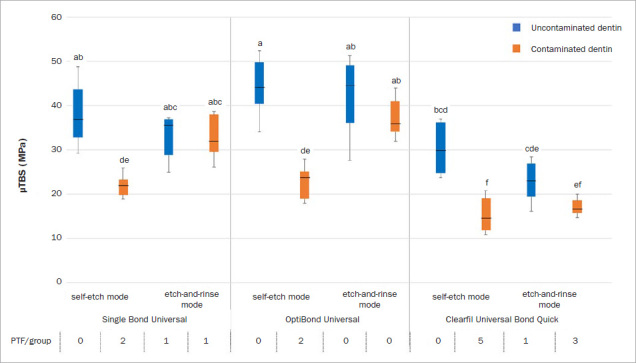
Box-plot of the microtensile bond strengths of adhesives to dentin in MPa and the number of PTF (pre-test failures) among the rectangular stick-shaped specimens from each experimental group.

The results of the three-way ANOVA and Scheffe’s test indicated significant effects of the types of universal adhesive (F = 97.758, p < 0.001), adhesive etching mode (F = 8.205, p = 0.005), and hemostatic agent contamination (F = 137.061, p < 0.001) on µTBS. The interactions between the tested factors were also significant, except for the interaction between all factors (p > 0.05). The bond strength data in MPa and the number of pre-test failures are presented in Fig 1.

The microtensile bond strength of the universal adhesives in the control group demonstrated no significant difference between etching modes. The highest bond strength was observed in the OPU group, followed by the SBU and CBQ groups. In contrast, in the CBQ group, bond strength was significantly lower compared with the OPU group in the same etching modes.

In the contaminated dentin groups, the µTBSs of the universal adhesives in ER mode were not significantly different compared with the control group. However, significantly lower universal-adhesive bond strengths were observed when bonded to contaminated dentin in SE mode. Compared within the same adhesive, the bond strength in ER mode was significantly higher than that in SE mode, except for CBQ, where no difference was detected between the two etching modes.

### Failure Mode Analysis

**Fig 2:**
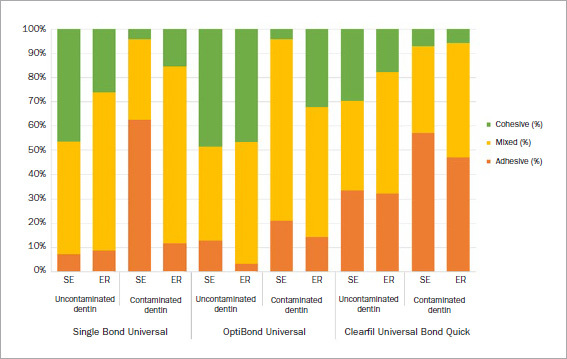
Failure modes in % (SE: self-etch mode, ER: etch-and-rinse mode).

The distribution of failure modes by group is illustrated in Fig 2. Failure modes in this study were classified into adhesive, mixed, and cohesive failure. Higher percentages of adhesive and mixed failure were observed when the adhesives were bonded to contaminated dentin. Adhesive failure was predominant in the SBU and CBQ groups when the adhesives were bonded to contaminated dentin in SE mode. In contrast, ~50% cohesive failure was observed in the OPU group when bonded to uncontaminated dentin.

### Resin-Dentin Interface Observation

**Fig 3:**
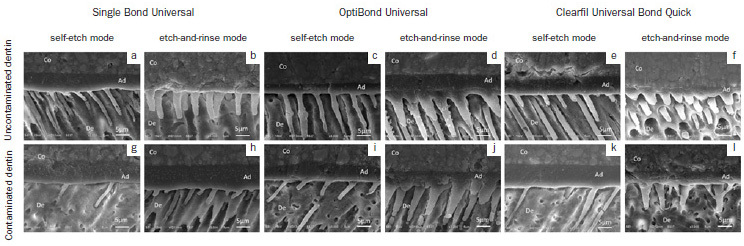
Representative SEM images of resin-dentin interface of universal adhesives (3000X). a) Single Bond Universal bonded to uncontaminated dentin in self-etch mode; b) Single Bond Universal bonded to contaminated dentin in self-etch mode; c) OptiBond Universal bonded to uncontaminated dentin in self-etch mode; d) OptiBond Universal bonded to contaminated dentin in self-etch mode; e) Clearfil Universal Bond Quick bonded to uncontaminated dentin in self-etch mode; f) Clearfil Universal Bond Quick bonded to contaminated dentin in self-etch mode; g) Single Bond Universal bonded to uncontaminated dentin in etch-and-rinse mode; h) Single Bond Universal bonded to contaminated dentin in etch-and-rinse mode; i) OptiBond Universal bonded to uncontaminated dentin in etch-and-rinse mode; j) OptiBond Universal bonded to contaminated dentin in etch-and-rinse mode; k) Clearfil Universal Bond Quick bonded to uncontaminated dentin in etch-and-rinse mode; l) Clearfil Universal Bond Quick bonded to contaminated dentin in etch-and-rinse mode. Co: composite, Ad: adhesive layer, De: dentin.

Representative SEM images of the resin-dentin interface of samples from each group are presented in Fig 3. A similar trend was observed among the different universal adhesives. The resin tags of the universal adhesives applied to uncontaminated dentin in SE mode had a long, cylindrical shape (Figs 3a, 3c, and 3e). However, the resin tags of the samples bonded to contaminated dentin were shorter and sparsely distributed by comparison (Figs 3 g, 3i, and 3k). In contrast, when the universal adhesives were applied in ER mode, similar characteristics were observed regardless of the adhesive used or the dentin substrate (Figs 3b, 3d, 3f, 3h, 3j, and 3l). The resin tags were conical and larger compared with those in the SE groups.

## DISCUSSION

The present study evaluated the µTBS of universal adhesives with different etching modes to human dentin contaminated with an aluminum chloride-containing hemostatic agent. The results demonstrated significantly lower bond strengths of the universal adhesives when bonded to hemostatic agent-contaminated dentin in SE mode. Therefore, the first and second null hypotheses were rejected. Furthermore, the CBQ bond strength was significantly lower compared with the other adhesives, so that the third null hypothesis was also rejected.

In this study, three universal adhesives were bonded either in SE mode or ER mode to uncontaminated dentin and hemostatic agent-contaminated dentin. Using universal adhesives allowed the direct effect of the etching modes to be evaluated. In the uncontaminated dentin group, the bond strength of the universal adhesives in SE and ER mode were similar. These results agree with those of previous studies.^7,8,21,30^ When bonding to contaminated dentin, using a universal adhesive in SE mode demonstrated inferior bonding performance. Similar results were found in previous studies using self-etching adhesives^4,9,10,16,19,26^ and universal adhesives in SE mode.^29^ This might be because the demineralization effect of the self-etching adhesive is weak. Therefore, the contaminant was not completely dissolved or eliminated using this adhesive.^2,5,16,19^ This concept is supported by the resin-dentin interface observation and failure mode analysis in the present study. In the SE group bonded to contaminated dentin, resin penetration was limited and the resin tags were relatively short. Moreover, the highest percentage of adhesive failure was demonstrated in this group, especially when SBU was used. It was reported that using a double application technique^6,12^ and prolonged adhesive application^16^ can improve the bond strength of a self-etch adhesive. Further studies should be performed with these techniques to determine whether they can restore the bond strength of contaminated dentin.

The results of the present study indicated that the hemostatic agent did not affect the dentin bond strength when the universal adhesives were bonded in ER mode. This is possibly due to the aggressive etching effect of phosphoric acid. This acid can demineralize the inorganic component of the dentin and possibly eliminate the negative effects of a hemostatic agent.^2,5,16^ This hypothesis is supported by our resin-dentin interface observation. Abundant conical resin tags were observed when the universal adhesives were bonded to contaminated dentin in ER mode, similar to those of uncontaminated dentin. These results are consistent with previous studies in which etch-and-rinse adhesives demonstrated better bonding performance to hemostatic agent-contaminated dentin.^14,16^ The results of this study suggest using a universal adhesive in ER mode when bonding to contaminated dentin.

The highest bond strength was achieved when OPU was used. The functional monomer in OPU is GPDM, whereas MDP is used in SBU and CBQ. Wang et al^31^ found that a GPDM-containing adhesive had a more aggressive etching effect. In addition, the hydrophilicity of GPDM is greater than that of MDP;^31^ thus, GPDM has higher wettability and can penetrate to deep dentin and form strong micromechanical interlocking.^31^ Furthermore, the molecular structure of GPDM contains two polymerizable groups, compared to MDP with only one polymerizable group.^31,33^ Therefore, a stronger resin matrix can be expected. However, the bond strength of OPU was not significantly different from that of SBU. This could be due to the Vitrebond copolymer in SBU that acts as another functional monomer and is responsible for its additional bond strength.^21,24,32^

According to Kuraray Noritake, a newly developed hydrophilic amide monomer in CBQ has a higher hydrophilic potential than 2-hydroxyethyl methacrylate (HEMA),^15,25^ resulting in higher wettability with a shorter application time. It was suggested to apply CBQ with a rubbing motion and proceed. Thus, the specific application time of CBQ was not indicated. In the present study, CBQ was applied for 5 s using the same application technique as with SBU and OPU. However, CBQ bond strength was significantly lower, especially when bonded to contaminated dentin in SE mode. This could be due to the shorter application time compared with the other adhesives at 20 s. It was reported that the shorter application time of the adhesive resulted in insufficient solvent evaporation and inferior bonding performance.^1,12,23,24^ Thus, the 5 s application time of CBQ in the SE group might result in insufficient smear-layer removal and lower bond strength to contaminated dentin. It was demonstrated that higher bond strength was achieved when the application time of CBQ was extended to 20 s.^1^ Similarly, the bond strength of CBQ to contaminated dentin might be increased with a longer application time.

## CONCLUSIONS

Contaminating dentin with a hemostatic agent adversely affects the microtensile bond strength of universal adhesives in self-etch mode. The bond strength can be restored by using a universal adhesive in etch-and-rinse mode.

## Clinical relevance:

Universal adhesives should be used with etch-and-rinse mode when bonded to dentin contaminated with a hemostatic agent.
